# Adverse events in thyroid surgery: observational study in three surgical units with high volume/year

**DOI:** 10.1186/s12893-021-01353-6

**Published:** 2021-09-25

**Authors:** Paolo Del Rio, Paolo Carcoforo, Fabio Medas, Elena Bonati, Tommaso Loderer, Margherita Koleva Radica, Piergiorgio Calò

**Affiliations:** 1 Unit of General Surgery, University Hospital of Parma, 14 Gramsci Road, Parma, Italy; 2 Unit of General Surgery, University Hospital of Ferrara, Cona, Italy; 3 Unit of General Surgery, University Hospital of Cagliari, Cagliari, Italy

**Keywords:** Vocal cord palsy, Thyroidectomy, Hypocalcemia, Postoperative bleeding, Dysphonia

## Abstract

**Background:**

Thyroid surgery, performed for benign or malignant pathologies, is one of the most frequently performed procedures and its frequency has even been increasing in recent years. Postoperative bleeding, recurrent laryngeal nerve (RLN) palsy, associated to dysphonia, dysphagia, dyspnea, and hypoparathyroidism represent the most fearful and common complications. We conducted a multicenter, observational study of retrospectively collected data in three high-volume referral centers, enrolling all patients undergone to thyroid surgery between January 2016 and December 2017 in Parma University Hospital, Cagliari University Hospital and Ferrara University Hospital.

**Materials:**

Patients were divided into five groups, differentiated thyroid carcinoma, medullary thyroid carcinoma, non-toxic benign pathology, hyperfunctioning benign pathology and NIFTP (Non-invasive Follicular Thyroid neoplasm with Papillary-like nuclear features). A follow up at 7 and 30 days was executed, evaluating the onset of paresthesia, dysphonia and dysphagia. A 6-month follow-up was conducted in cases of early complications.

**Results:**

Totally, 1252 patients were eligible for the study: 907 female and 345 male, with a female to male ratio of 2.6:1 and an average age of 53.428. Total thyroidectomy was performed in 1022 cases, lobectomy in 230. After 6 months we recorded paresthesia in 0.5%, dysphonia in 1.8% and dysphagia in 0.5%.

**Conclusion:**

Our study confirms once again that a share of morbidity escapes the possibilities of prediction and control by the operator, depending on patient anamnestic, pathological or anatomical factors.

## Introduction

Thyroid surgery, performed for benign or malignant pathologies, is one of the most frequently performed procedures and its frequency has even been increasing in recent years with the increased incidence of differentiated thyroid cancer.

The incidence of post thyroidectomy complications has steadily decreased in recent decades, thanks to technical improvements and technological advances, reaching the lowest rates in high-volume referral centers [1–3].

Postoperative bleeding, recurrent laryngeal nerve (RLN) palsy, associated to dysphonia, dysphagia, dyspnea, and hypoparathyroidism represent the most fearful and common complications. Capsular dissection, visual identification and intraoperative nerve monitoring have reduced the incidence of RLN injury to 1–2% in tertiary referral centers, showing that in some cases neuronal damage must be consider an inevitable complication rather than a "surgical error" [4, 5].

Therefore, permanent complications can result in a marked deterioration in the patient's quality of life, affecting personal, social and working life. This is frequently cause of surgical malpractice claims.

For this reason, many studies have tried to identify predictive factors for the possible onset of complications, including characteristics of patient, of underlying disease or of surgical technique. In our study, we wanted to overcome the population variability deriving from different geographic areas and the variability in the surgical habits of different surgical teams, including patients undergoing surgery in three high-volume referral centers.

## Methods

We conducted a multicenter, observational study of retrospectively collected data in three high-volume referral centers, enrolling all patients undergone to thyroid surgery between January 2016 and December 2017 in Parma University Hospital, Cagliari University Hospital and Ferrara University Hospital.

Totally, 1252 patients were eligible for the study. Collected data included patient demographics, anamnesis and clinical-instrumental-laboratory data, such as thyroid ultrasound pattern, preoperative diagnosis by ultrasound-guided fine needle aspiration (FNA) reported according to the Bethesda System for Reporting Thyroid Cytopathology [6], preoperative TSH, PTH and calcemic, X-ray trachea / CT neck, preoperative fibroscopy.

Intraoperative data included type of surgery performed (total thyroidectomy or lobectomy), number of parathyroid glands seen during surgery, lymph node dissection, use of intermittent intraoperative neuromonitoring (i-IONM), hemostatic agents, intraoperative administration of corticosteroids.

During hospitalization, bleeding, paresthesia, dysphonia, dysphagia, dyspnea, first day calcium value, first day PTH value, hospital therapy with intravenous calcium, oral calcium or corticosteroids, length of hospitalization were recorded. Paresthesia, dysphonia, dysphagia and dyspnea are subjectively reported by the patient during the clinical visit in case, respectively, of bilateral tingling in the extremities or perioral, voice alterations, alterations in swallowing, in particular fluids and breathing difficulties.

After hospitalization, we assessed any therapy with calcium and vitamin D, post-operative fibroscopy, definitive histopathological diagnosis, tumor size, angiovascular invasion, infiltration of perithyroid tissues, metastatic lymph nodes, presence of parathyroid glands in the specimen, radiometabolic therapy and speech therapy.

Based on definitive histological diagnosis, patients were divided into 5 groups, differentiated thyroid carcinoma, medullary thyroid carcinoma, non-toxic benign pathology, hyperfunctioning benign pathology and NIFTP (Non-invasive Follicular Thyroid neoplasm with Papillary-like nuclear features).

A follow up at 7 and 30 days was executed, through an outpatient visit, evaluating the onset of paresthesia, dysphonia and dysphagia. A 6-month follow-up was conducted in cases of early complications.

Only adult patients were included in the study, and patients previously undergone to thyroid surgery or presenting vocal cords pathologies on preoperative fibroscopy were excluded, as potential confounders.

Univariate analysis was conducted to evaluate the influence of demographic, preoperative, intraoperative, and pathological factors on postoperative complications, as potential effect modifier. The studied factors included, depending on the complication under consideration, age, sex, high blood pressure, use of anticoagulant/antiaggregant, type of intervention, intraoperative administration of corticosteroids, use of suction drainages, use and findings of IONM, use of hemostatic agents, pathological diagnosis, postoperative calcemic and presence of parathyroid glands in the specimen. In case of missing data, patient was excluded from the analysis. Chi-squared test and Student’s t-test were used for categorical data and for continuous variables, respectively. Variables < 0.100 in the univariate analysis were considered significant and were then included in the multivariate analysis. Logistic regression analysis was used to identify independent risk factors of postoperative complications. Results were considered statistically significant if p-value was < 0.05. All analyses were carried out using IBM SPSS Statistics, version 19.

## Results

Patients included in the study were 1252, 907 female and 345 male, with a female to male ratio of 2.6:1 and an average age of 53.428. Considering the site of the intervention, 474 procedure were executed at Parma University Hospital, 504 at Cagliari and 274 at Ferrara University Hospital. Patients with missing data were previously excluded from the study.

Medium preoperative TSH was 1.562 uU/ml and calcium was 9.425 mg/dl. Demographic and preoperative laboratory tests are reported in Table 1.

**Table 1:** Demographic and preoperative laboratory tests

	Mean	Std error	95% Confidence interval for mean		5% Trimmed mean	Median	Std deviation	Minimum	Maximum
			Lower bound	Upper bound					
Age	53.428	0.4012	52.641	54.215	53.579	54.000	14.1954	18.00	89.00
Preoperative TSH	1.562	0.0396	1.484	1.639	1.452	1.309	1.2442	0.0	7.0
Preoperative serum calcium	9.425	0.0144	9.397	9.453	9.412	9.400	0.4517	8.0	12.1

Preoperative cytological examination by FNA was performed in 715 patients and the result was reported according to the Bethesda System for Reporting Thyroid Cytopathology [6]. Results are reported in Table 2. Patients without suspected nodularity at preoperative thyroid ultrasound examination, such as diffuse non-toxic goiter and colloidocystic goiter or Graves Basedow's disease, did not undergo cytological examination.

**Table 2:** Preoperative cytological examination by fine needle aspiration (FNA), reported according to Bethesda System

		Frequency	Percent
FNA	Thyr 1	26	2.1
	Thyr 2	195	15.6
	Thyr 3	89	7.1
	Thyr 4	205	16.4
	Thyr 5	63	5.0
	Thyr 6	137	10.9
	Total	715	57.1
Non executed		537	42.9
Total		1252	100.0

Total thyroidectomy was performed in 1022 cases, lobectomy in 230; in 130 patients, a lymph node dissection was also performed.

I-IONM was used in 958 procedures, according to the habits of the different operating units. In fact, in two centers it was routinely used while in one it was used only in complex selected cases, such as neoplasms suspected for perithyroid infiltration, voluminous goiters, reoperations or laterocervical lymphadenomies. In 24 cases a loss of signal was recorded. Overall, 8.9% of patients underwent postoperative fibroscopy, which was routinely performed by a surgeon while it was prescribed by other operators only in case of persistent dysphonia 30 days after surgery. Speech therapy was necessary in 3.4% of cases.

Complications arising on day 1, day 7, day 30 and at 6 months were then evaluated. On day 1, bleeding was found in 3.2% of patients, paresthesia in 6.4%, dysphonia in 4.8%. Calcemic in the first postoperative day showed an average value of 8.47 mg / dl, ranging from a minimum of 5 mg / dl to a maximum of 12 mg / dl. On the 7th day, paresthesia were found in 1.7% of cases, dysphonia in 4.4%, dysphagia in 0.9%. On day 30, paresthesia were found in 1.3% of patients, dysphonia in 3.8% and dysphagia in 0.3%. After 6 months we recorded paresthesia in 0.5%, dysphonia in 1.8% and dysphagia in 0.5%.

The relationship between the type of surgery (lobectomy or total thyroidectomy) and the onset of complications at the various established follow-up steps (1st, 7th, 30th day and 6 months) was analyzed. (Table 3).

**Table 3:** Postoperative complication and type of intervention

		Hemithyroidectomy (%)	Total thyroidectomy (%)	P value
Bleeding	Day 1	1.9	3.7	0.214
Parestesia	Day 1	0.9	8.0	0.000
	Day 7	1.4	1.9	0.700
	Day 30	1.4	1.3	0.269
	6 Months	0.5	0.6	0.900
Dysphonia	Day 1	1.9	5.9	0.021
	Day 7	6.3	3.7	0.129
	Day 30	5.8	3.0	0.052
	6 Month	2.9	1.3	0.139
Dysphnea	Day 7	1.0	0.9	0.273
	Day 30	0.5	0.2	0.483
	6 Month	1.0	0.4	0.320

Bleeding on day 1 occurred in 1.9% of patients undergoing lobectomy and in 3.7% of patients undergoing total thyroidectomy, with no statistically significant difference. Paresthesia occurred on day 1 in 0.9% of patients undergoing lobectomy, while in patients undergoing total thyroidectomy they occurred in 8%, thus showing a statistically significant correlation (p = 0.000). The correlation between dysphonia in day 1 and type of intervention also proved to be statistically significant, occurring in 5.9% of patients who underwent total thyroidectomy (5.9%), while only in 1.9% of lobectomy cases. Regarding the complications arising on day 7, 30 and after 6 months (paresthesia, dysphonia, dysphagia) no significant correlations emerged; however, there is a higher percentage of dysphonia among lobectomies (6.3%) compared to total thyroidectomies (3.7%), in all follow-up phases. Nevertheless, among patients undergoing lobectomy who presented dysphonia on day 7, a loss of NIM signal was recorded in 76.9%, there was no loss of IONM signal in 7.7% and the IONM has not been used in 15.4%

As this is a retrospective study, it was not possible to trace which of these patients were originally enrolled for a total thyroidectomy and therefore how many procedures became lobectomies due to intraoperative NIM signal loss, according to the indications of the two stage thyroidectomy [7]. In the pre-IONM era, these cases would have relapsed into the group of patients undergoing total thyroidectomy.

Considering the loss of NIM signal and the onset of dysphonia on the entire sample of patients, only one patient presented this association on the 1st postoperative day, while we found a statistically significant association on the 7th (p = 0.000) and 30th day (p = 0.000) and after 6 months (p < 0.001).

It was then assessed whether the onset of paresthesia could correlate with the calcemic values detected on the 1st postoperative day. Paresthesia were recorded in 6.48% of cases in the first postoperative day. Oral calcium supplementation during hospitalization was introduced only in case of onset of paraesthesia.

In patients without paresthesia on day 1, the calcium has an average value of 8.53 mg / dl, with a minimum of 7 mg / dl and a maximum 12 mg / dl, while in patients who presented paresthesia the average calcium is attests to a significantly lower value of 7.59 mg / dl on average, with a minimum value of 5 mg / dl and a maximum of 9 mg / dl. A correlation between the calcemic value in the first day and the development of paresthesia was highlighted in all the stages considered (p = 0.000 in the 1st, 7th and 30th day, p = 0.004 at 6 months).

A correlation was sought between the onset of paresthesia and the presence of parathyroid glands in the surgical specimen. However, no statistically significant relationships emerged. In fact, only 9.2% of patients with presence of parathyroid glands at the definitive histological examination, presented paresthesia on the first day, on the 7th day 2.5% and on the 30th day 1.7%; on the other hand, among patients negative for the presence of parathyroid glands, paresthesia were recorded in 1.6% in day 7 and in 1.3% in 30th.

After 6 months, among patients in whom the presence of parathyroid glands was found, there was not even one case of paresthesia, while 0.6% of those who did not have parathyroid glands accidentally removed at the final histological examination were recorded.

The mean length of hospitalization was 2.459 days, showing a minimum of 1 day and a maximum of 14 days. Patients who experienced bleeding on day 1 had a hospital stay of 4.174 days while those who did not have bleeding reported a mean hospital stay of 2.076 days. Even the onset of paresthesia in the first day minimally prolonged hospitalization, with an average duration of 2.625 days compared to 2.265 days for patients without paresthesia. Similarly, the development of dysphonia in day 1 led to an average hospital stay of 3.730 days compared to 2.062 days for patients without dysphonia. Analyzing data with the Kruskall-Wallis test, a statistically significant difference was highlighted between the hospitalization of patients who presented at least one of the complications considered in the 1first day and the duration of hospitalization (p < 0.000 in all cases).

Finally, we evaluated the possible association between thyroid disease, divided into 5 diagnostic categories (differentiated carcinoma, medullary carcinoma, non-hyperfunctioning benign disease, hyperfunctioning benign disease, NIFTP) and the onset of complications.

The analysis found only a statistically significant correlation between the hyperfunctioning benign disease and the onset of bleeding and paresthesia in first postoperative day. No association was recorded between thyroiditis on pathology specimen and postoperative complications. Full results are shown in Table 4.

**Table 4:** Postoperative complication and histological diagnosis

		CDT (%)	CM (%)	NT-BTD (%)	T-BTD (%)	NIFTP (%)	P value
Bleeding	Day 1	3.4	0.0	2.0	15.8	0.0	0.014
Parestesia	Day 1	8.4	0.0	4.0	16.4	0.0	0.001
	Day 7	2.2	0.0	1.3	0.0	0.0	0.856
	Day 30	1.9	0.0	0.8	0.0	0.0	0.936
	6 Months	0.3	0.0	0.5	0.0	0.0	0.991
Dysphonia	Day 1	4.3	0.0	5.3	10.5	0.0	0.717
	Day 7	4.7	0.0	4.4	0.0	0.0	0.867
	Day 30	3.1	0.0	4.4	0.0	0.0	0.951
	6 Month	1.3	0.0	2.1	0.0	0.0	0.877
Dysphnea	Day 7	0.9	0.0	0.5	5.9	0.0	0.555
	Day 30	0.3	0.0	0.0	0.0	0.0	0.862
	6 Month	0.3	0.0	0.5	0.0	0.0	0.990

*CDT* differentiated thyroid carcinoma, *CM* medullary carcinoma, *NT-BTD* non-toxic benign thyroid disease, *T-BTD* toxic benign thyroid disease, *NIFTP* non-invasive follicular thyroid neoplasm with papillary-like nuclear features

**Fig. 1:**
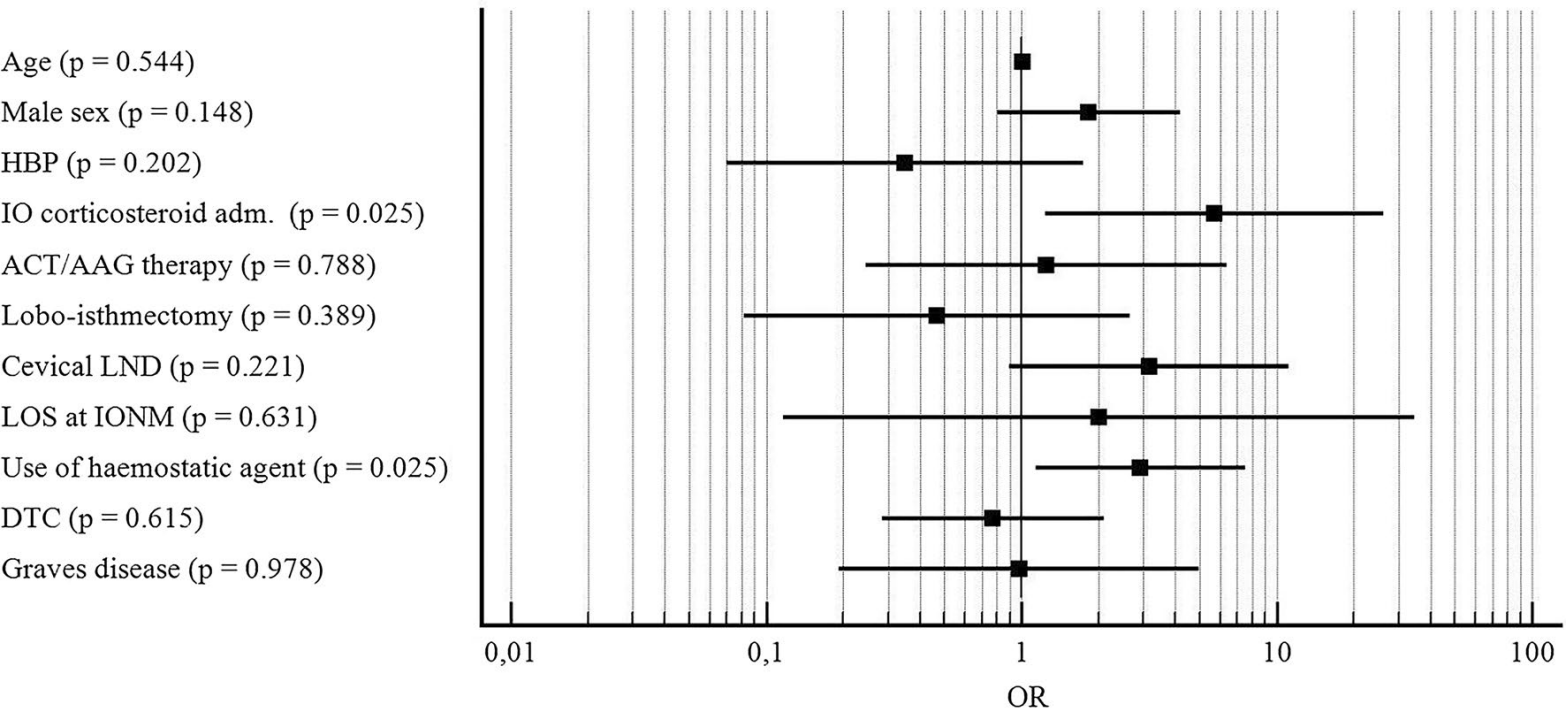
Forest plot reporting results of multivariate analysis considering postoperative dysphonia as dependent variable. *OR* odds ratio, *HBP* high blood pressure, *IO* intraoperative, *ACT/AAG* anticoagulant/antiaggregant, *LND* lymph node dissection, *LOS* loss of signal, *IONM* intraoperative nerve monitoring, *DTC* differentiated thyroid carcinoma. X axis is reported in logarithmic scale

At multivariate analysis, intraoperative corticosteroid administration (OR = 5.682; CI: 1.2329—26.1859; p = 0.025) and the use of haemostatic agent during surgery (OR = 2,928; CI: 1.1383 – 7.5345; p = 0.025) were found as independent risk factors for postoperative dysphonia (Fig. 1).

**Fig. 2:**
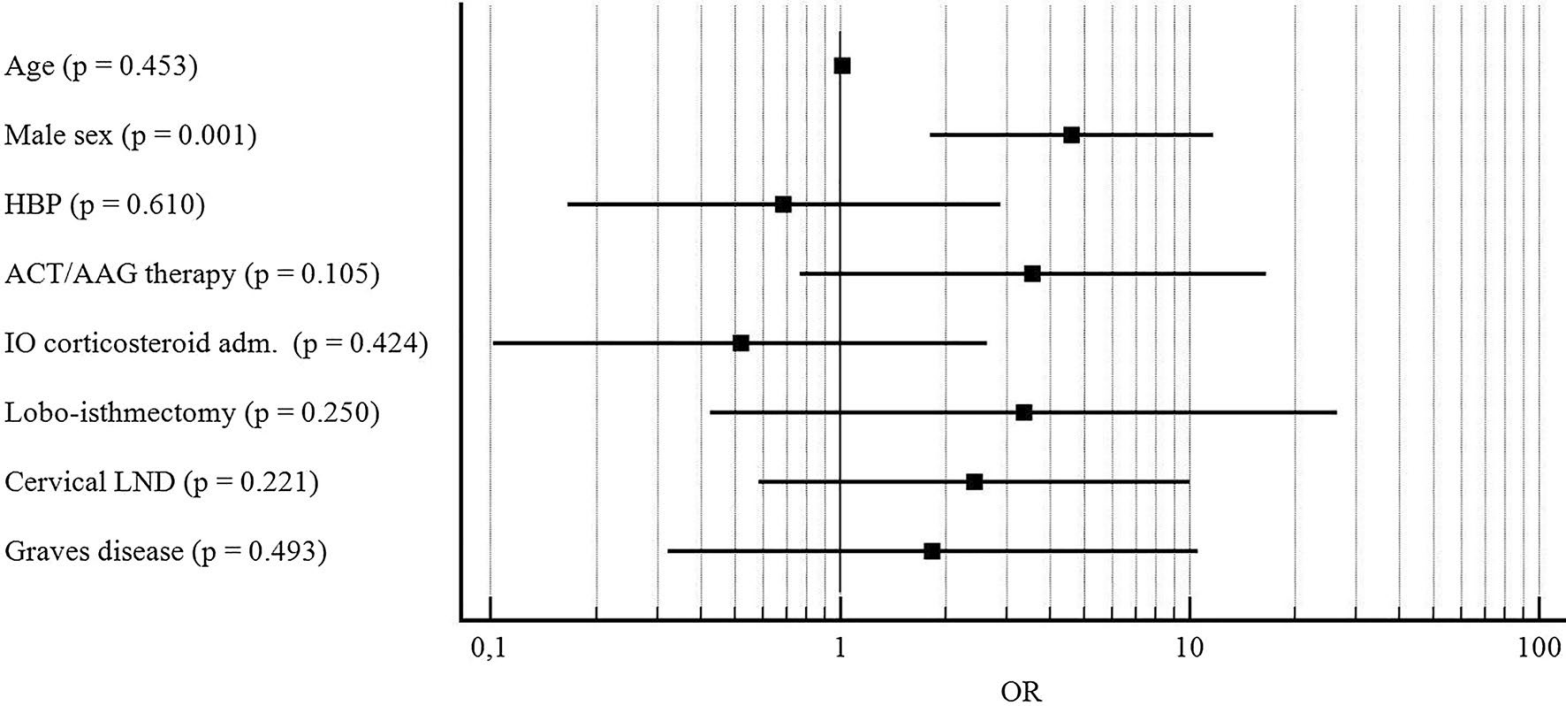
Forest plot reporting results of multivariate analysis considering postoperative bleeding as dependent variable. *OR* odds ratio, *HBP* high blood pressure, *ACT/AAG* anticoagulant/antiaggregant, *IO* intraoperative, *LND* lymph node dissection. X axis is reported in logarithmic scale.

**Fig. 3:**
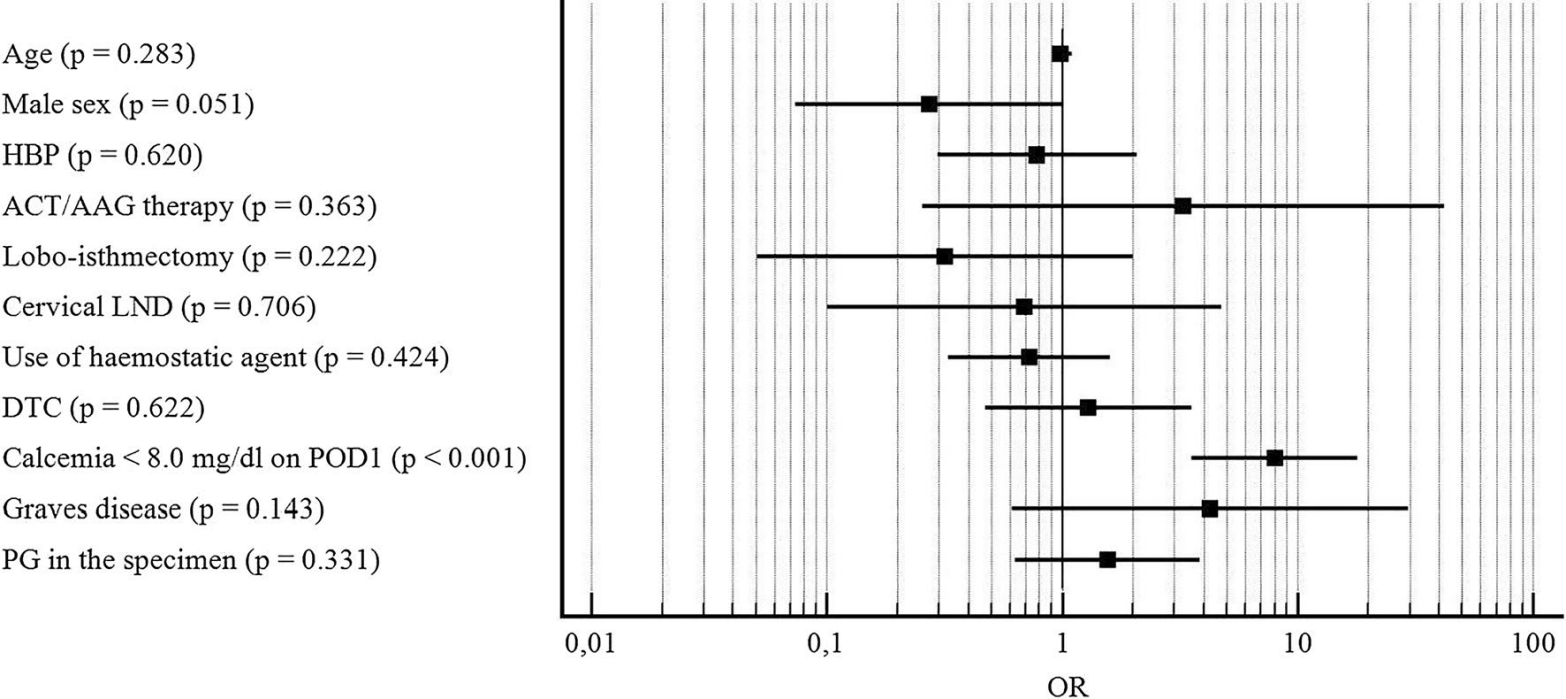
Forest plot reporting results of multivariate analysis considering postoperative paraesthesia as dependent variable. *OR* odds ratio, *HBP* high blood pressure, *ACT/AAG* anticoagulant/antiaggregant, *LND* lymph node dissection, *POD1* postoperative day 1, *PG* parathyroid glands. X axis is reported in logarithmic scale.

Male sex (OR = 4.606; CI: 1.8132–11,7053; p = 0.001) was found as independent predictive factor for postoperative bleeding (Fig. 2); and a postoperative calcemia < 8.0 mg/dl (OR = 7.994; CI: 3.555–17.9763; p < 0.001) was identified as independent predictive factor for paraesthesia (Fig. 3).

## Discussion

Thyroid surgery, although with a low incidence, can present some specific postoperative complications that have a strong impact on the patient's quality of life and are often a source of medico-legal dispute.

The incidence rates reported in the literature are highly variable and primarily influenced by the surgeon's experience and by the volume of the center. All studies agree that operations performed by experienced surgeons and in high volume centers (> 100 thyroidectomies / year) show a lower complication rate and a shorter hospital stay [8].

In particular, it has been shown that the length of stay and complications are more determined by the surgeon experience than by hospital volume, which does not have a consistent association with the results [3].

Some study described also a threshold (> 25 total thyroidectomies/y) that identify a high-volume thyroid surgeon and it is associated with improved patient outcomes [9].

In our multicenter study, Parma, Cagliari and Ferrara University Hospital are high-volume referral centers and high-volume thyroid surgeons have executed all procedures included. This led to the finding of a low complication rate, uniform among the three centers despite differences in surgical practice, first in the use of the IONM. In particular, the average incidence of definitive dysphonia settles at 1.8% in our series, with literature reported rate from 0.7 to 5.65% [10–13].

Direct visualization of the RLN still remains the cornerstone of avoiding damage, while the non-visualization during dissection causes the highest risk for nerve palsy occurrence. No consensus exist regarding the utility of IONM. In particular, different studies have compared neural visualization and dissection alone to identification plus intraoperative use of NIM, without the evidence of any statistically significant difference [14–17]. Anaway, the routinary use of NIM has the enormous advantage to avoid bilateral palsy in case of loss of signal on the initial site, forcing to stop the operation [18–20]. After RLN palsy, the recovery of the nerve at one year is expected to be as high as 95% when the anatomic integrity is confirmed during the procedure. Consequently, the non-visualization of the nerve during dissection represents a striking risk factor for permanent damage [10].

Additionally, it has been reported that a large thyroid mass predicts a poor recovery after RLN injury, but this do not represent an independent risk factor for complications [17].

In our study, the use of hemostatic agents during surgery was found as independent risk factors for postoperative dysphonia, probably due to a greater use of coagulation instruments too in a more bloody operating field during procedure, with greater risk of thermal nerve injury, or due to the development of postoperative adhesions, which alter normal pharyngo-laryngeal mobility.

The development of muscle adhesions, which impair mobility, can also causes an associated dysphagia. This complication has a more complex pathogenesis, related to trauma from orotracheal intubation, postoperative tissue swelling and presence of pre-existing functional gastroesophageal disorders. For the most part, however, these are transient disturbances, which recede spontaneously [21–23].

Minor surgical trauma and reduced postoperative pain could be responsible in part for the better vocal and swallowing outcomes found in patients operated with MIVAT technique [24, 25].

Thyroiditis on pathology specimen is not associated with increased postoperative complications in our study, while the presence of thyroid hyperfunction is associated with both an increased rate of bleeding and transient hypoparathyroidism with paresthesia, as confirmed in literature [26–29].

Paraesthesias is the most frequent postoperative complication, related to the lowering of serum calcium levels in the postoperative period. It can be attributed to a number of causes, which can also coexist, such as hemodilution associated with surgical stress, decreased renal tubular reabsorption, vitamin D deficiency and acute increase in calcitonin levels. The main cause of hypocalcemia after thyroidectomy appears to be hypoparathyroidism [30–32].

Lymph node dissection and female gender are associated with this complication, as previously suggested in case series, but parathyroid function recovery is not affected by these factors [33–36].

Considering the lack of association between the accidental removal of parathyroid glands and the onset of paresthesias, which however correlate with the levels of calcium in the first postoperative day, our study seems to suggest that postoperative hypocalcemia depends on numerous surgical and personal factors of the patient, difficult to predict.

## Conclusions

The possible complications resulting from thyroid surgery have a strong impact on patients and are frequently the cause of medico-legal disputes. Despite the technological improvement, the volume of the dedicated thyroid surgeon and the meticulous dissection and visualization of the intraoperative RLN, remain the gold standards for decreasing the incidence of complications. In our study, compared to an equal volume of centers involved and equal experience of surgeons, the different technologies or protocols routinely applied did not influence the outcomes, as some centers routinely use the I-IONM while others occasionally and different devices are used during intervention, based on surgeons’ habits. Overall, low complication rates were recorded, however not zero.

This confirms once again that a share of morbidity escapes the possibilities of prediction and control by the operator, depending on patient anamnestic, pathological or anatomical factors.

## Data Availability

The datasets used and analysed during the current study are available from the corresponding author on reasonable request.
